# Pilonidal sinus disease on the rise: a one-third incidence increase in inpatients in 13 years with substantial regional variation in Germany

**DOI:** 10.1007/s00384-021-03944-4

**Published:** 2021-05-16

**Authors:** Christina Oetzmann von Sochaczewski, Jan Gödeke

**Affiliations:** 1 Klinik und Poliklinik für Kinderchirurgie, Universitätsmedizin der Johannes-Gutenberg-Universität, Mainz, Germany; 2 Sektion Kinderchirurgie der Klinik und Poliklinik für Allgemein-, Viszeral-, Thorax- und Gefäßchirurgie, Universitätsklinikum Bonn, Venusberg-Campus 1, D-53127 Bonn, Germany; 3 Klinik und Poliklinik für Kinderchirurgie, Dr. von Haunersches Kinderspital der Ludwig- Maximilians-Universität, München, Germany

**Keywords:** Routinely collected health data, Health services research, Age groups, Rate of inpatient episodes, Epidemiology, Pilonidal sinus

## Abstract

**Purpose:**

Collective evidence from single-centre studies suggests an increasing incidence of pilonidal sinus disease in the last decades, but population-based data is scarce.

**Methods:**

We analysed administrative case–based principal diagnoses of pilonidal sinus disease and its surgical therapy between 2005 and 2017 in inpatients. Changes were addressed via linear regression.

**Results:**

The mean rate of inpatient episodes of pilonidal sinus disease per 100,000 men increased from 43 in 2005 to 56 in 2017. In females, the mean rate of inpatient episodes per 100,000 women rose from 14 in 2005 to 18 in 2017. In the whole population, for every case per 100,000 females, there were 3.1 cases per 100,000 males, but the numbers were highly variable between the age groups. There was considerable regional variation within Germany. Rates of inpatient episodes of pilonidal sinus disease were increasing in almost all age groups and both sexes by almost a third. Surgery was dominated by excision of pilonidal sinus without reconstructive procedures, such as flaps, whose share was around 13% of all procedures, despite recommendations of the national guidelines to prefer flap procedures.

**Conclusion:**

Rates of inpatient episodes of pilonidal sinus disease in Germany rose across almost all age groups and both sexes with relevant regional variation. The underlying causative factors are unknown. Thus, patient-centred research is necessary to explore them. This should also take cases into account that are solely treated office-based in order to obtain a full-spectrum view of pilonidal sinus disease incidence rates.

## Introduction

The incidence of pilonidal sinus disease is on the rise globally [1]. Incidences are often reported from selected cohorts, either the military or in the age group known to have high incidences of the disease [2, 3], whereas population-based incidences are seldom reported [4, 5]. Consequently, the same—outdated—study reporting an incidence rate based on a single institution with its surrounding population gets cited again and again [6], even in national guidelines [7] and Cochrane collaboration reviews on pilonidal sinus disease [8]. We therefore explored German administrative data in order to describe population-based rates of inpatient episodes separated by different age groups and investigated potential regional differences within a country.

## Methods

We obtained aggregated datasets from the Statistisches Bundesamt (Federal Statistics Office) including principal diagnoses and procedures of the German Modification of the International Classification of Diseases – Version 10 for the years 2005 to 2017. These datasets were analysed for cases with a principal diagnosis of pilonidal sinus disease with (ICD-10-GM L05.0) and without (ICD-10-GM L05.9) concurring abscess. In addition, the therapy was evaluated for pilonidal sinus excision without a plastic reconstruction (OPS 5-897.0) and with any plastic reconstruction (OPS 5-897.1), such as midline closure, a transposition flap or a rotational flap, e.g. the Karydakis flap, the Dufourmentel flap or the Limberg repair. The regional distribution of diagnoses and cases was based on the location of the hospital. Rates of inpatient episodes were calculated by division of principal diagnoses by the official population number at the reporting day of the Statistisches Bundesamt and provided in cases per 100,000 people. Rates of inpatient episodes were calculated separately for the age groups provided within the data. Of note, the administrative database is derived from hospital reimbursement statistics and covers cases, but not individual patients. Detailed properties and pitfalls of this data have been discussed elsewhere [9]. Studies using administrative data are exempt from ethical approval, because the case-based data cannot be traced back to the individual patient [10].

Statistical analyses were conducted using R (RRID: SCR_001905) (version 3.5.3) with its generic stats4 package [11], if not stated otherwise. Differences over time were assessed using linear regression, which has been deemed suitable for these type of data [12–15]. The necessary prerequisites of linear regression—normality of errors and homogeneity of variances—were tested using the Kolmogorov-Smirnov test and the Breusch-Pagan test from the olsrr package (version 0.5.3) [16] supported by visual analysis of QQ plots. Confidence intervals for point estimates were calculated using the *t* distribution via the Rmisc package (version 1.5) [17, 18]. Shape data—to visualise regional differences in the rate of inpatient episodes—were obtained from the Bundesamt für Kartographie und Geodäsie (Federal Agency for Cartography and Geodesy) [19] as described before [15]. Colour palettes from the viridis package (version 0.5.3) were used to draw colour-blind friendly figures [20].

## Results

**Fig. 1:**
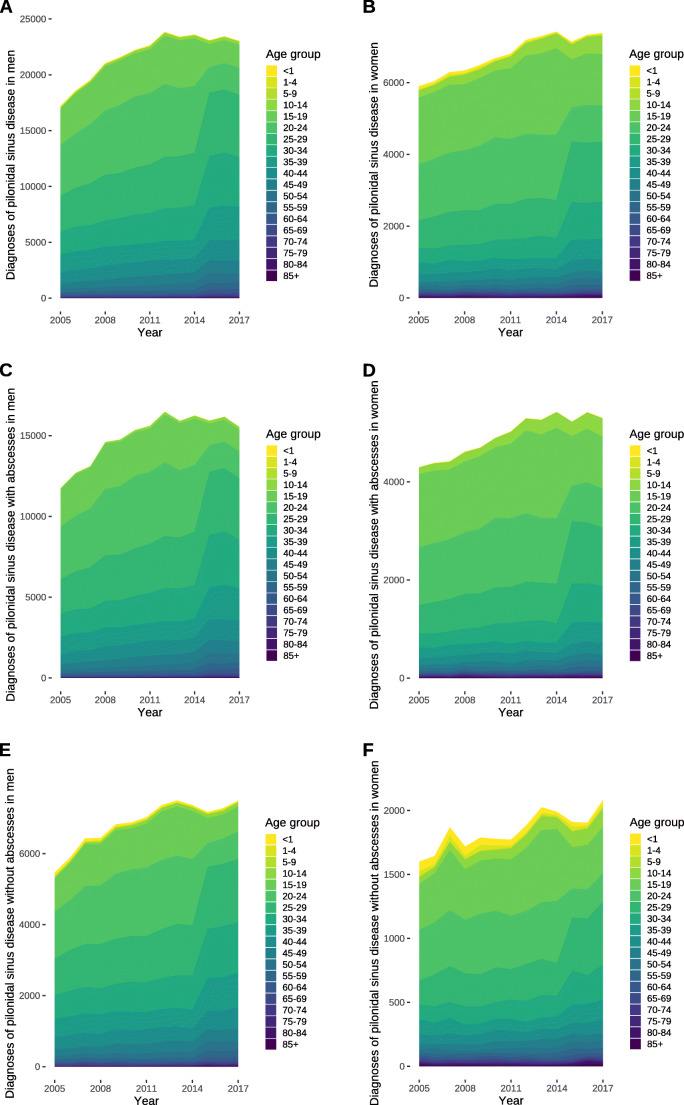
Annual diagnoses of pilonidal sinus disease in Germany. Data represents absolute numbers of diagnoses per year in Germany separated by age groups. **a** Cumulative number of diagnoses of pilonidal sinus disease in men. **b** Cumulative number of diagnoses of pilonidal sinus disease in women. **c** Number of diagnoses of pilonidal sinus disease in men that manifest with an abscess. **d** Number of diagnoses of pilonidal sinus disease in women that manifest with an abscess. **e** Number of diagnoses of pilonidal sinus disease in men that occur without an abscess. **f** Number of diagnoses of pilonidal sinus disease in women that occur without an abscess

The cumulative number of diagnoses increased by 476 (95% CI, 299–655) cases per year (*P* < 0.0001) in males (Fig. 1a), of which 329 (95% CI, 193–464) were cases with an abscess (Fig. 1c) and 147 (95% CI, 99–195) without an abscess (Fig. 1e) (both *P* < 0.0001). In females, the cumulative number of diagnoses increased by 133 (95% CI, 106–159) cases per year (*P* < 0.0001) (Fig. 1b), of which 101 (95% CI, 78–124) (Fig. 1d) occurred with a concurring abscess (*P* < 0.001) and 32 (95% CI, 19–44) did not (*P* = 0.0002) (Fig. 1e).

**Fig. 2:**
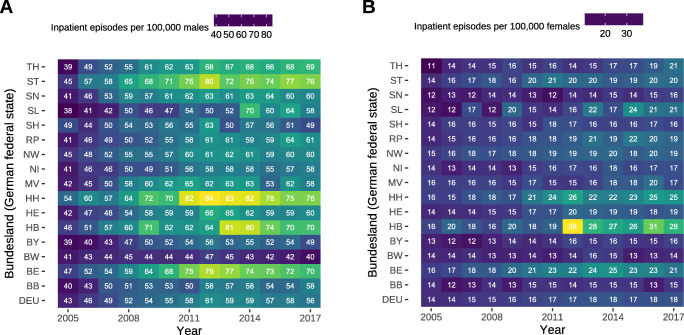
Rates of inpatient episodes in Germany per *Bundesland* (German federal state). Data represents rates of inpatient episodes per 100,000 persons in Germany as a whole and in the respective *Bundesland* (German federal state). Rates are coloured according to their level, and the respective number represents the annual rate of inpatient episodes per 100,000 persons to ease interpretation. **a** Rates of inpatient episodes per 100,000 males. **b** Rates of inpatient episodes per 100,000 females. DEU = Germany, BB = Brandenburg, BE = Berlin, BW = Baden-Württemberg, BY = Bavaria, HB = Bremen, HE = Hesse, HH = Hamburg, MV = Mecklenburg Western Pomerania, NI = Lower Saxony, NW = Northrhine Westphalia, RP = Rhineland Palatinate, SH = Schleswig-Holstein, SL = Saarland, SN = Saxony, ST = Saxony-Anhalt, TH = Thuringia

**Fig. 3:**
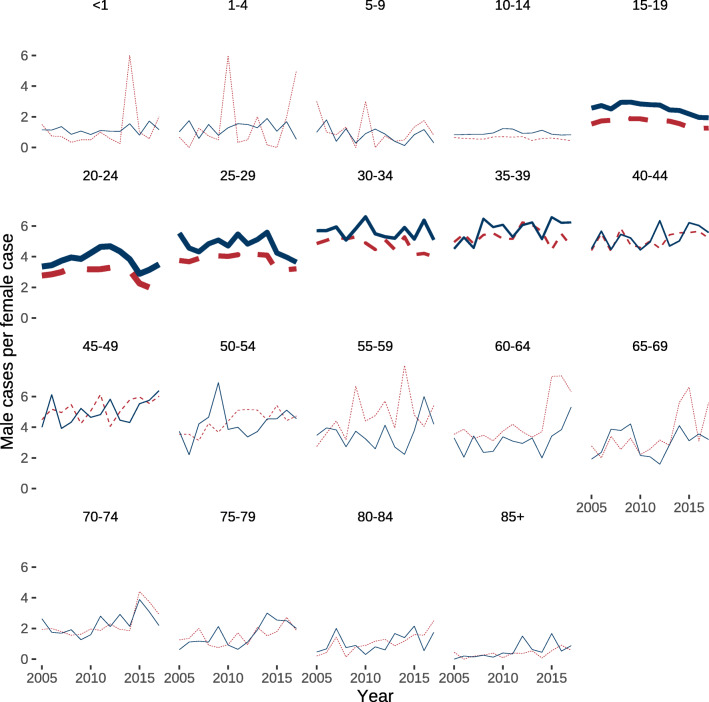
Sex ratio of cases of pilonidal sinus disease. Data represents the distribution of cases between both sexes by the number of male cases per female case. Blue (solid) lines represent cases without and red (dashed) lines cases with a concurring abscess. The line width is proportional to the number of cases in the respective age group

This overall increase in cases did correspond to an increasing rate of inpatient episodes of 1.16 (95% CI, 0.62–1.7) cases per 100,000 males (*P* < 0.0001) (Fig. 2a) and 0.33 (95% CI, 0.25–0.41) cases per 100,000 females (*P* < 0.0001) (Fig. 2b). Across all age groups, there were 3.1 cases per 100,000 males for every case per 100,000 females, which did not change during the study period (Fig. 3). However, this relationship between the sexes was highly different between the age groups (Fig. 3).

**Fig. 4:**
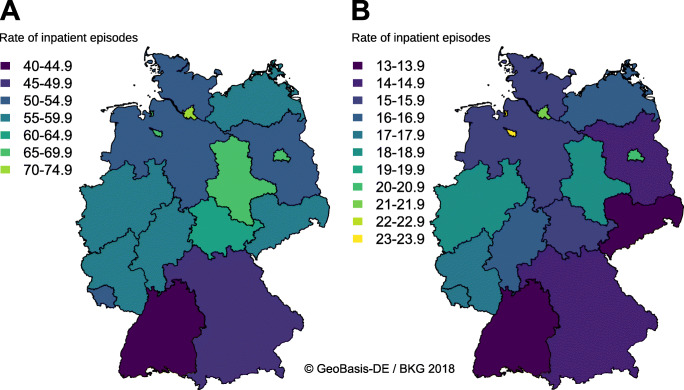
Regional variation of rates of inpatient episodes of pilonidal sinus disease in Germany. Data represents rates of inpatient episodes per 100,000 persons in the respective *Bundesland* (German federal state). **a** Rates of inpatient episodes per 100,000 males. **b** Rates of inpatient episodes per 100,000 females

Rates of inpatient episodes in many *Bundesländer* (German federal states) were close to the German annual mean increase per 100,000 males (Fig. 2). We found considerable regional differences between the *Bundesländer* (German federal states), which ranged from a minimum in Baden-Württemberg to a maximum in Hamburg in males (Fig. 4a) and from a minimum in Saxonia to a maximum in Bremen in females (Fig. 4b).

**Fig. 5:**
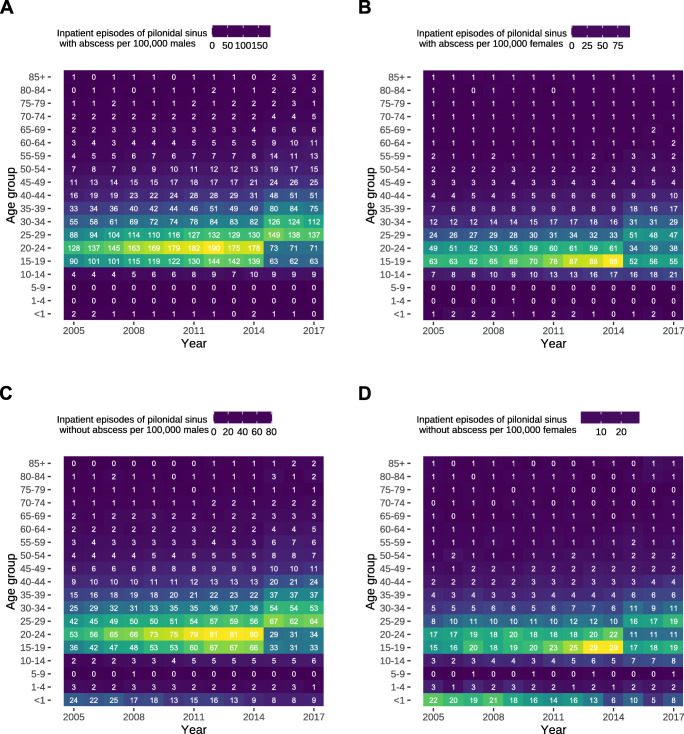
Rates of inpatient episodes of pilonidal sinus disease with and without concurring abscess in men and women of different age groups. Data represents rates of inpatient episodes per 100,000 persons. Rates are coloured according to their level, and the respective number represents the annual rate of inpatient episodes per 100,000 persons to ease interpretation. **a** Rates of inpatient episodes of pilonidal sinus with concurring abscess per 100,000 males. **b** Rates of inpatient episodes of pilonidal sinus with concurring abscess per 100,000 females. **c** Rates of inpatient episodes of pilonidal sinus without concurring abscess per 100,000 males. **d** Rates of inpatient episodes of pilonidal sinus without concurring abscess per 100,000 females

Among the different age groups, a relevant reduction in rates of inpatient episodes could only be observed in the first year of life for cases without an abscess with a yearly decrease of 1.44 (95% CI, 1.1–1.79; *P* < 0.0001) cases per 100,000 males (Fig. 5c) and an annual decrease of 1.4 (95% CI, 1.06–1.73; *P* < 0.0001) cases per 100,000 females (Fig. 5d). The largest contribution of the increasing incidences for cases with concurring abscess was due to the age groups with beginning adolescence—in females those above the age of 10 years and in males above the age of 15 years—with a maximum increase of 5.51 (95% CI, 3.81–7.22; *P* < 0.0001) cases per 100,000 males in the age group between 30 and 34 years, whereas the only age groups with constant rates of inpatient episodes were those up to 9 years of age (Fig. 5a).

Interestingly, we found a steep increase in cases per 100,000 males and females in the age groups of both 15 to 19 and 20 to 24 years of age with and without abscesses until 2014, which experienced a sharp decline afterwards—more pronounced in males than in females—that could only be described using quadratic linear regression (Fig. 5). Beyond this unusual observation, the pattern of increasing rates of inpatient episodes for female cases with concurring abscess was similar to those in males—although the maximum increase occurred in those between 25 and 29 years of age with 2.01 (95% CI, 1.29–2.74; *P* < 0.0001) per 100,000 females annually—with the exception of a missing increase beyond retirement age (Fig. 5b). The development in cases without abscesses mirrored their counterparts with abscesses (Fig. 5c, d).

**Fig. 6:**
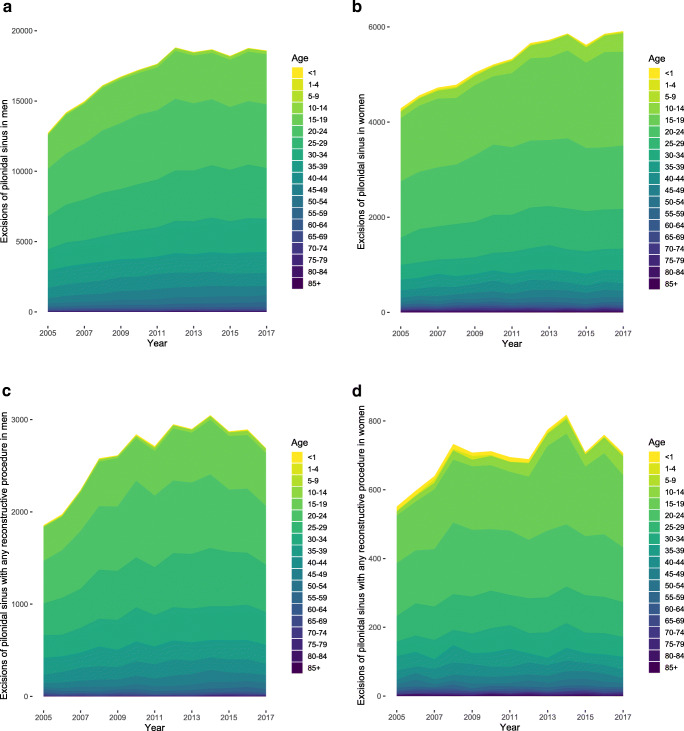
Surgical excision of pilonidal sinus with and without reconstructive procedure in men and women of different age groups. Data represents cumulative number of procedures separated by sex and age groups per year. **a** Excision of pilonidal sinus in males without reconstructive procedure. **b** Excision of pilonidal sinus in females without reconstructive procedure. **c** Excision of pilonidal sinus in males followed by a reconstructive procedure. **d** Excision of pilonidal sinus in females followed by a reconstructive procedure

**Fig. 7:**
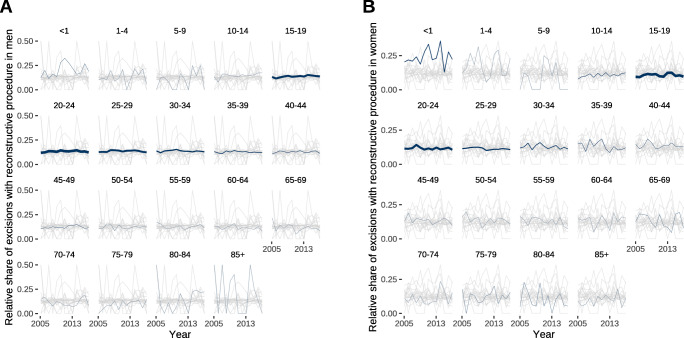
Relative share of pilonidal sinus excisions followed by any reconstructive procedure by age group. Data represents the fraction of all pilonidal sinus excisions that were combined with any reconstructive procedure such as transposition or rotation flaps. The blue line represents the data for the respective age group, whose line width is proportional to the number of procedures in this age group. **a** Proportion of reconstructive procedures in pilonidal sinus excision in males. **b** Proportion of reconstructive procedures in pilonidal sinus excision in females

The annual increase in pilonidal sinus excisions in men was 460 (95% CI, 317–602; *P* < 0.0001) and 137 (95% CI, 112–161; *P* < 0.0001) in women (Fig. 6a, b). Likewise, excisions followed by any reconstructive procedure did increase by 78 (95% CI, 40–117; *P* = 0.0009) per year in men and 13 (95% CI, 5–22; *P* = 0.0058) annual procedures in women (Fig. 6c, d). The proportion of simple excisional procedures and those with a reconstructive procedure did not change over time (Fig. 7). The relative share of excisions of pilonidal sinus followed by reconstructive procedures was similar between 10 and 15% in almost all age groups (Fig. 7). Those age groups with relatively low absolute numbers of procedures (Fig. 6) had a higher variability in relative shares of reconstructive procedures (Fig. 7).

## Discussion

Global incidence rates of pilonidal sinus disease are on the rise [1, 21], and its burden of disease may exceed those of inguinal hernias in the severely affected age groups [22]. However, incidence rates of pilonidal sinus disease are often described using decades-old data [6] that may not reflect today’s true incidence rate due to the rapid growth since then. Reliable, preferably large-scale, data are necessary not just to raise awareness among care providers but also to guide and evaluate healthcare policy decisions [23]. We therefore used German administrative data to report population-based rates of inpatient episodes among all age groups and both sexes.

Our dataset not only revealed much higher overall rates of inpatient episodes than the well-cited report from the 1990s [6]—reflecting the increasing incidence reported before [1]—but, more importantly, also contradicts the well-accepted [3, 7, 24, 25] notion that patients above the age of 30 would seldom be affected by pilonidal sinus disease. This was particularly the case in men, which had a burden of disease comparable to mean rates of inpatient episodes of their sex until their mid-40s, but to a lesser extent in women. Their rates of inpatient episodes began to rise in the age group above 10 years of age, whereas this more pronounced increase in rates of inpatient episodes occurred in the age group of 15 to 19 years in males. This might be an effect of the earlier onset of puberty in females, which has been described to be an important factor in the onset of pilonidal sinus disease, based on the fact that rates of inpatient episodes in girls started to increase in the age group above 10 years, whereas the rates of inpatient episodes in this age group remained relatively low in boys [26]. This may be supported by the ratio of cases between the sexes: There were always more cases per 100,000 males than per 100,000 females except for the age group between 10 and 14 years. However, cases per 100,000 females were also more frequent in the age group between 5 and 9 years, which are unlikely to be linked to the onset of puberty.

Previous research demonstrated considerable worldwide variation in the burden of disease in females [27]. Likewise, our dataset also revealed considerable regional variation in rates of inpatient episodes, not only between continents but also within Germany for both men and women. The underlying reasons have already been unclear for the worldwide regional variation [27], which is also true for the regional variation within Germany, which may not be explained using administrative data.

Not in concordance with the literature was the unexpected high rate of inpatient episodes in the first year of life, although decreasing over time, and rates of inpatient episodes comparable to octogenarians in those children up to the age of 4 years: According to the paediatric surgical literature—irrespective of anecdotal decades-long observations of a single paediatric surgeon [28] or a systematic review [29]—cases outside adolescence should be a rarity, but were quite prevalent within the German administrative data. This type of data is, however, unable to explain this observation, but it should prompt paediatric surgical departments to investigate their cases of pilonidal sinus disease in infants and toddlers.

The data on treatment shows that the vast majority of performed procedures are excisions of pilonidal sinus, whereas reconstructions via flap procedures are used in around 13% of cases. The codes may not differentiate between primary suturing, shown to be associated with a higher risk of recurrence, compared to reconstructive procedures and healing by secondary intention [1, 8, 30]. It has been suggested that the low number of reconstructive procedures used in real-world data is the result of a combination of a lack of formal training, particularly in flap procedures, and a lack of expertise due to many surgeons performing low-volume pilonidal sinus surgery [24]. The administrative data thus indicate that the recommendations of the S3 guideline on pilonidal sinus disease to favour flap procedures [31, 32] have not been implemented in daily practice.

Although German administrative data have been used to examine the relationship between volume and outcome before, this has been done using the hard endpoint of mortality [33, 34]. As this endpoint is an absolute exception following excision of a pilonidal sinus, it may not be used for the evaluation of pilonidal sinus disease. Due to the case-based nature of the data, the same patient may be included in the data more than once, e.g. if he was treated for a recurrence in the same year or even a complication of the primary procedure that was treated in a different hospital, which constitutes a major limitation. In the absence of a specific code for a re-do procedure, which has been used in other diseases to evaluate the recurrence or failure rate of the primary surgery [35], the answer to this question could, again, only be solved by using patient-centred data instead of case-based that we did use in the present report. Another limitation of the regional coding is the potential distortion of aggregated data, because it assigns the location code according to the location of the hospital instead of the patient’s place of residence. However, it is well known that for minor and office-based procedures, patients prefer a hospital that is very close to their place of residence [36], so that the expected distortion of regional differences might be rather small and exert relevant influence on the distribution of cases between the *Bundesländer* (German federal states).

The most relevant advantage of administrative data is their completeness due to the compulsory participation by hospitals and the resulting completeness of inpatient data [9]. This completeness does, however, only apply to cases that were treated on an inpatient basis. Contrary to diagnoses of hip fractures in adults [37] or hypertrophic pyloric stenosis in infants [15], operations for pilonidal sinus disease are on the list of procedures that may be conducted either as inpatient or office-based procedures and their methods—such as pit picking—that are specifically used for office-based procedures [38], as recommended by the S3 guideline for pilonidal sinus disease [31, 32]. While it is difficult to conduct less complex inguinal hernia repair cost-covering on inpatients [39], the financial spread between the inpatient and office-based operation is much smaller for pilonidal sinus disease [40] and, thus, the incentive to conduct surgery preferably on inpatients is smaller than that for other diagnoses.

Financial aspects may also explain the specific development of inpatient episodes in patients aged 15 to 24, which have a maximum number of episodes in 2014 that drop sharply afterwards: There is a relevant coincidence between this sharp decline in inpatient episodes and the development of the legal concept of the virtual efficient alternative (*fiktives wirtschaftliches Alternativverhalten*) [41]. This concept prohibits reimbursement by the health insurance system if there is an equally suitable alternative that is more cost-efficient than the one that was chosen by the healthcare provider [41]. Although there is no relationship between duration of pilonidal sinus disease and increasing numbers of pilonidal sinus [42], it is tempting to speculate—due to a paucity of data on this aspect [31, 32]—that a higher share of patients might have a more limited disease or have not undergone a preceding treatment and would thus be eligible [31, 32] for office-based treatment and avoid reimbursement cuts. It is, however, impossible to investigate this point, because only a single cumulative number of office-based procedures in Germany is reported, but not separated by diagnoses or procedures [43].

Another issue is that the German system of reimbursement via diagnoses-related groups has only been introduced in 2005, which might be the cause of a distortion of results: The vast majority of healthcare providers did work outside the pilot hospitals and thus had to familiarise themselves with the new system, potentially causing inaccuracies. Looking at the rates of inpatient episodes in men in Germany, the steepest increase in rates was between 2005 and 2008, which could have been influenced by the adaptation phase of healthcare providers. In order to assess this potential bias, we recalculated the rates of inpatient episodes for pilonidal sinus excluding the years 2005 to 2008. We found no relevant differences in the regression coefficients: Instead of being smaller, they tended to be larger if the early years of the German system of diagnoses-related groups were not included, except for those men aged 15 to 24, in whose age groups the coefficients of the quadratic regression were smaller. There has been only one result that was not statistically significant anymore, which pertains to men at the age of 85 and above. We can therefore assume that the effects of this adaptation phase to the reimbursement system via diagnoses-related groups were rather small.

Nevertheless, administrative data offers the advantage of describing population-based rates of inpatient episodes to their compulsory nature and thereby sheds light on aspects that may have been missed by the usual case series and offers the opportunity of being relatively simple compared to expensive and time-consuming data collection on several study sites in patient-based research [44]. However, true incidence rates may not be calculated, because cases treated on an outpatient basis are missing. Numbers more close to reality—as they include information on procedures conducted on outpatients in hospitals and are patient- instead of case-based [14]—might be obtained from healthcare claims data from the German statutory health insurances.

In conclusion, we reported detailed population-based rates of inpatient episodes for pilonidal sinus disease separated by sex and age. These rates of inpatient episodes are substantially increasing in all age groups except children and have considerable regional variation, whose causes are entirely unclear. In Germany, treatment of pilonidal sinus disease is largely conducted without reconstructive procedures despite their potentially recurrence-preventing effect. Patient-centred research is necessary to explore underlying causative effects, which are entirely unclear at present.

## Data Availability

The data supporting the findings of this study are available from the Statistisches Bundesamt (German federal statistics office).
